# The Gas Sensing Drone with the Lowered and Lifted Measurement Platform

**DOI:** 10.3390/s23031253

**Published:** 2023-01-21

**Authors:** Andrzej Szczurek, Dawid Gonstał, Monika Maciejewska

**Affiliations:** 1 Faculty of Environmental Engineering, Wroclaw University of Science and Technology, 50-370 Wrocław, Poland; 2 Wroclaw Centre for Networking and Supercomputing, Wroclaw University of Science and Technology, 50-370 Wrocław, Poland

**Keywords:** gas sensing, air quality, environmental monitoring, UAV

## Abstract

A serious factor that limits the environmental applications of drones is the disturbance of the air pollution concentration field by the drone propulsion system. This work presents a gas-sensing drone offering measurements that are unaffected by this phenomenon. The novel development was based on the idea that, during measurements, the sensing device should be spatially separated from a zone influenced by the drone’s rotors. To attain this goal, special equipment was designed that allows one to undock and lower the sensing device for measurement, lift it and dock for flight. The field experiments demonstrated the full functionality of the developed system and its superiority compared to a sensing platform mounted at the bottom of the drone. Higher measurement sensitivity and resolution were attained by lowering the sensing platform to the measurement point. This solution minimizes the rotor flow effect, ground effect, and pollution concentration field flattening. The test in real conditions confirmed that the designed construction assures drone stability. The presented technology may be an important step in developing effective mobile measurement tools that allow one to reach poorly accessible or dangerous places and perform measurements at a low cost and with high efficiency.

## 1. Introduction

The rapid development of drone technology has resulted in its application for the detection, identification, and monitoring of chemical substances releases into the atmosphere [1]. The use of unmanned aerial vehicles (UAVs) as a platform for carrying the chemical detector offers significant advantages. The most important one is measurements in harsh and poorly accessible environments without hazard to human operators [2,3]. Other benefits are rapid screening and time-efficient supervision of large areas and detecting pollution events [4]. Maps overlaying with 3D data and including them in Geographic Information Systems is possible [5].

Currently, several crucial factors are limiting the effective performance of drones in environmental applications. The problems result, e.g., from the interferences that are produced by the propulsion system of the drone [6,7]. The vortexes and fast downward flow of air caused by the rotation of the drone’s propellers can significantly disturb the spatial and temporal distribution of the chemical composition of air [8]. It is the reason for the inaccurate detection of hazardous substances released into the atmosphere and misreading their real concentrations. In such circumstances, gas sensing provides rather useless results. The elimination of these limitations is a challenging task. Different strategies are proposed to overcome the problems. There are various options considered for the location of the sensing device on the drone, e.g., radial positioning [9], mounting between drone rotors [10], or fixing it on the top of the drone [11]. Some of them consist in limiting the scope of measurement tasks, e.g., to the vertical profiling of the atmosphere during upward flights exclusively [11,12]. Some rely on sampling probes extended sideways, beyond the anticipated field of disturbances [13]. Others try to find the best flight trajectory [14]. There are also developments aimed at integrated sample collection only [15,16]. 

This work aims to present a low-cost gas-sensing drone that offers measurements that are unaffected by the air turbulences generated by propellers.

In our study, it was assumed that a sufficiently long distance between the inlet of a sampling system and the drone’s propellers may reduce the interferences with the measurement signal resulting from the movement of the rotor blades. Such an assumption means that the sensing device should be spatially separated from a drone’s propellers during measurements. In other words, sensors cannot be mounted on board or near UAVs. To fulfill this requirement, construction ensuring an appropriate distance between sensors and rotors is needed. In addition, the applied construction cannot affect the flight of the drone. This goal may be attained by specially designed equipment that is attached to a drone and allows for lowering and lifting the sensing device. Such an approach allows for the extension of the measurement range of gas-sensing drones beyond the downward air flow and turbulence zone caused by the rotation of rotors. To verify this hypothesis there was a gas-sensing drone developed, characterized by a specific design and mode of operation, see Section 8. 

The developed solution is novel and has several advantages. (1) The downwash effect, ground effect, and pollution concentration field flattening were minimized. The reliability of the measurement was improved regarding the level as well as temporal/spatial variation of pollution. (2) Innovative systems for measurement platform lowering/lifting and docking were developed. They allow for the stable flight of the drone and it hovering while the sensing platform is lowered/lifted or remains lowered at the measurement point. (4) Several measurement points may be served during one flight with one set of batteries. Currently, the time required to lower and lift the measurement platform is no longer than 10% of the drone’s operating time at fully charged batteries. (5) The measurement may be performed at locations that are difficult to access or dangerous. The measurement platform can be delivered to places where the drone itself cannot fly in, thanks to the possibility of its lowering and lifting. (6) The presented solution minimizes the influence of pollution on the drone mechanism. Thus, its protection from harsh environments is achieved. Only the sensing device has to be exposed to pollution.

The sensing drone was tested in field conditions. It should be noted that the full functionality of the system has been confirmed during field experiments, including unfavorable weather vacations.

The rest of the paper is structured as follows. Section 2 presents the architecture of the novel gas-sensing drone with the lowered and lifted measurement platform. Its subsections are dedicated to the drone itself (Section 2.1), gas sensing platform (Section 2.2), the equipment for lowering and lifting the measurement module (Section 2.3), docking system (Section 2.4), and radio communication (Section 2.5). In particular, Section 2.2 provides the detailed characteristics of the gas sensing platform, including elements such asair intake and gas filters (Section 2.2.1), three-way valve (Section 2.2.2), set of sensors and measurement chamber (Section 2.2.3), air-extracting pump (Section 2.2.4), gas transfer line (Section 2.2.5), and microcontroller (Section 2.2.6). Section 3 briefly describes how the sensing drone operates. The field tests of the system were extensively characterized in Section 4. Section 5 presents the results of the measurement experiments. Their discussion is included in Section 6. The main conclusions are formulated in Section 7. 

## 2. Architecture of the Gas Sensing Drone with the Lowered and Lifted Measurement Platform

**Figure 1:**
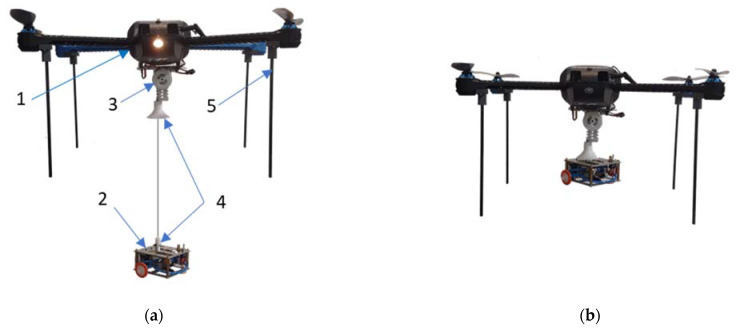
Gas sensing drone, when the measurement platform is: (**a**) lowered; (**b**) docked under the drone. (1) UAV (quadrocopter); (2) Gas sensing platform; (3) Equipment for lowering and lifting the measurement module; (4) Docking system; (5) Landing gear.

The gas-sensing drone is presented in Figure 1. It comprises (1) an UAV (drone); (2) a gas sensing platform, and (3) equipment for lowering and lifting the measurement module.

The drone is controlled via a 9-channel remote/radio controller (RC) transmitter programmed for three flight modes: altitude hold mode (STD)—altitude hold mode and manual flying using the controller; loiter mode (LTR)—altitude hold mode and automatic positioning using global positioning system (GPS) signal, and mission mode (AUTO)—automatic mode for autonomous missions.

The two transmission channels of the RC transmitter, CH8, and Ch6, control the operation of the device for lowering and lifting the measurement platform by generating the corresponding pulse width modulation (PWM) signal at the output of the aircraft’s flight controller. The CH7 channel settings generate a signal in the range of 991 to 2015 depending on the position of the bi-stable button specifying the downward or upward direction in which the measurement platform is to move. Channel CH8 generates a signal in the range of 992 to 2016 depending on the position of another button, which controls the automatic process of lowering/lifting the measuring platform in a monostable manner. The button released starts an automatic sequence of breaking and finally stopping the direct current (DC) motor which powers the device for lowering and lifting the measuring platform.

With this solution the pilot can control the flight of the drone and the position of the measurement platform at the same time with great precision. This ensures the stable and safe operation of the entire system. For example, the developed solution allows for full control, during a sudden swing of the lowered measuring platform. The pilot can control the distance of the measurement platform from the UAV in real-time, so he can change the force vector causing the swaying of the measurement platform by shortening or unwinding the holding line at the end to which the platform is attached. In extreme cases, the system allows one to correct the position of the drone with the simultaneous lifting of the measurement platform in such a way that the pilot safely docks the swayed element, minimizing the risk of crashing the drone by losing control over it.

The sensing platform is equipped with an independent system and two-way radio frequency (RF) data transmission with a range identical to the RC transmitter controlling the drone. Such a solution makes it possible to control the measurement process and for a user equipped with an RF received connected to a laptop, tablet, or phone to view the collected data in real time. Real-time analysis and communication with the pilot can improve the quality of the acquired measurement data.

### 2.1. UAV (Drone)

A quadrocopter equipped with 850 kV motors was selected as the drone. It was used to carry a gas-sensing platform and auxiliary equipment to lower and raise the platform. Quadrocopters attracted our attention because of their vertical landing, good maneuverability, high agility, and takeoff capabilities, as well as their simple design, cost-effectiveness, and small size. In our setup, we used a quadcopter from 3DR Iris, 3D Robotics, Berkeley, CA, USA, see Figure 1. The drone was controlled remotely. Control commands were issued by the operator at a distance of up to 1 km. Pixhawk Flight Controller v2.4.5, 3D Robotics, Berkeley, CA, USA, was used to control the flight. It is a controller designed as "open source", allowing the user to freely configure and modify the drone’s control system. The mission planning system allows for the configuration of autonomous flight along with the operation of peripheral devices without pilot control, in our case a sensor measurement system. The Ublox NEO-7N GPS, U-blox, Thalwil, Switzerland, module was used for flight positioning.

The weight of the drone, including the battery, was 1282 g. The total weight of the measurement system (drone and the measurement equipment including the winch) was 1642 g. The weight of the auxiliary measurement system including the winch did not exceed the lifting capacity limit of 400 g specified by the manufacturer of the 3DR Iris quadcopter. The drone was powered by a 5 Ah 11.1 V lithium-polymer battery, which, according to the manufacturer, should be sufficient for about 16–22 min of flight. During the tests, the maximum flight time was 9 min, leaving about 30–40% of the battery undischarged, depending on weather conditions. The maximum flight speed did not exceed 10 km/h.

During the measurements, the drone was controlled in LTR mode, providing constant altitude and automatic positioning using GPS signals.

To assure adequate space for the measurement system, between the drone body and the ground, a special landing gear was prepared, see Figure 1. It was made of lightweight and strong fiberglass tubes. They had a length of 215 mm, an inner diameter of 3 mm, and walls of 1 mm thickness. The landing gear was attached to the quadrocopter’s four arms via dedicated adapters made of polyvinyl chloride (PVC).

### 2.2. Gas Sensing Platform

**Figure 2:**
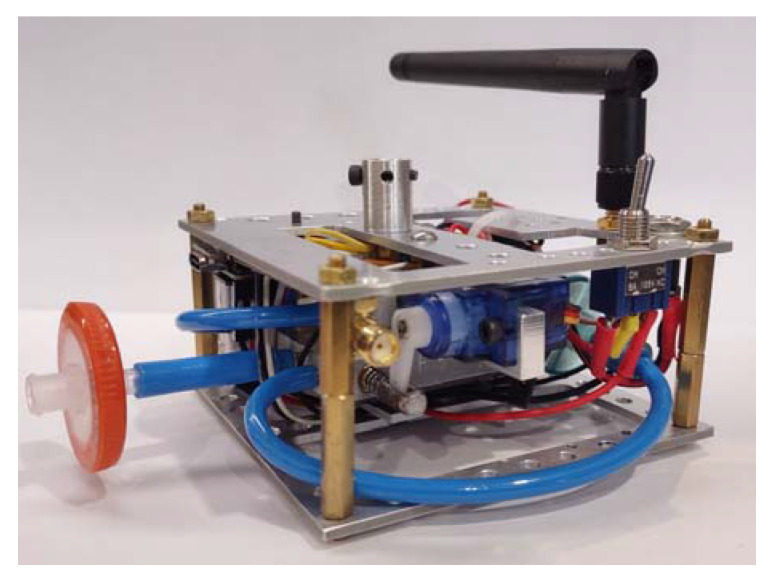
The view of the gas sensing platform.

The developed gas sensing platform was dedicated to measuring air pollution. The view of the sensing platform is shown in Figure 2. It was characterized by a compact design. Its size was 90 mm × 90 mm × 60 mm. The design was compatible with drone size, shape, and mobility. The elements used to build the measurement platform were lightweight, low-cost, and robust to be successful in use. The construction was based on a lightweight, damage-resistant aluminum frame. The weight of the entire sensing unit, including the power supply section and the battery, was 203 g. The sensory platform was equipped with a quick coupler, which allowed the element to be quickly connected to the holding line.

**Figure 3:**
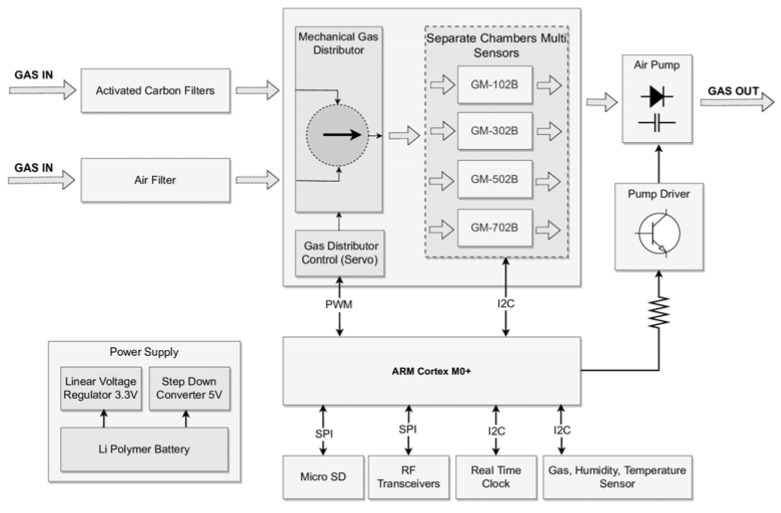
The schema of the gas sensing platform.

This module was designed to accomplish the following functions: (1) air sampling; (2) conditioning of a test air; (3) generation and measurement of sensor responses to a test gas; (4) signal conditioning; (5) data acquisition and transmission; (6) preparation of reference/cleaning gas, and (7) sensor regeneration. The functions of the gas sensing platform determined its construction. As shown in Figure 3, the gas sensing platform consisted of the following components: air intake; two filters; a three-way valve; a measurement chamber with a set of sensors inside; microcontroller; an air extracting pump; a gas transfer line; lithium polymer batteries, and enclosure with a mounting plate.

The specific modular design of the measurement platform made it possible to change the system configuration while maintaining a small size and low weight. All digital devices mounted on the platform were powered by 3.3 V, obtained through linear voltage stabilization of a 7.4 V li-pol battery with a capacity of 700 mAh. A step-down boost converter with an output voltage of 5 V was used to power the air pump and the servo controlling the three-way valve.

The average current consumption was 180 mAh, when the air pump was running at the default level of 66% of its power, while the instantaneous current consumption during data transfer and valve repositioning did not exceed 390 mAh.

All modules of the measurement platform communicated with the microcontroller through digital and analog interfaces. Using the serial peripheral interface (SPI), the microcontroller communicated with the RF module and transmitted real-time data directly to the user over a distance of up to 1 km. In addition, for security, the collected data were stored on a micro secure digital (SD) card on the measurement platform using the SPI bus with a specific chip select (CS) pin assigned to the module. The PWM signal was used to control the position of the three-way valve, managing the gas flow.

The digital-to-analog converter (DAC) output of the microcontroller, via a BC337 transistor, Diotec Semiconductor, Heitersheim, Germany changed the speed of the DC motor the air pump was equipped with, regulating the gas flow through the measurement system. 

The Real-Time Clock (RTC) module supplemented any information stored on the micro-SD card with the current date and time. It was equipped with a DS1307 chip, Adafruit Industries, New York, NY, USA and a CR1220 backup battery, Baltrade, Gdansk, Poland. It communicated with the microcontroller via inter-integrated circuit (I2C) protocol buses. 

To control the height of the measurement platform, a BME680 multifunctional environmental sensor from Bosch Sensortec, Reutlingen, Germany [17] was added. It was mounted outside the sensing device. It communicated with the microcontroller using an I2C interface.

The dedicated computer application featured by the graphical user interface (GUI) was developed. It allows full control of the measurement process, data collection, and graphical visualization of measurement data in real time using dynamic line graphs. The application is compatible with Windows.

#### 2.2.1. Air Intake and Gas Filters

The air intake diameter was 4 mm. The device air inlet was connected with two kinds of filters. Air filter 1 consisted of a membrane and it was intended to remove the particulate matter from the air at the intake. Air filter 2 was based on activated carbon and it was intended for the preparation of a cleaning/reference gas. This operation relied on the removal of volatile organic compounds (VOCs). The preparation of the cleaning gas was required for the regeneration of semiconductor gas sensors dedicated to VOCs measurement. Regarding reference gas, the filter was capable of removing VOCs from the air. Therefore, the reference measurement for VOCs sensors could be done in any circumstances, i.e., regardless of the composition of air at the intake. However, air filter 2 was not capable of removing NO_2_ and CO from the air passing through it. Therefore, the reference measurement for NO_2_ and CO sensors had to be performed in a location where the air was clean as compared to the air composition at the actual measurement point. During measurement, the test air was directed from intake via air filter 1 to the measurement chamber. During sensor cleaning, for reference measurement, the air was directed through air filter 1 and 2 in sequence, before reaching the measurement chamber. It was assumed that during measurement, air intake was positioned at the level of a measurement point, beyond the air turbulence zone, caused by the drone rotor.

#### 2.2.2. Three-Way Valve

**Figure 4:**
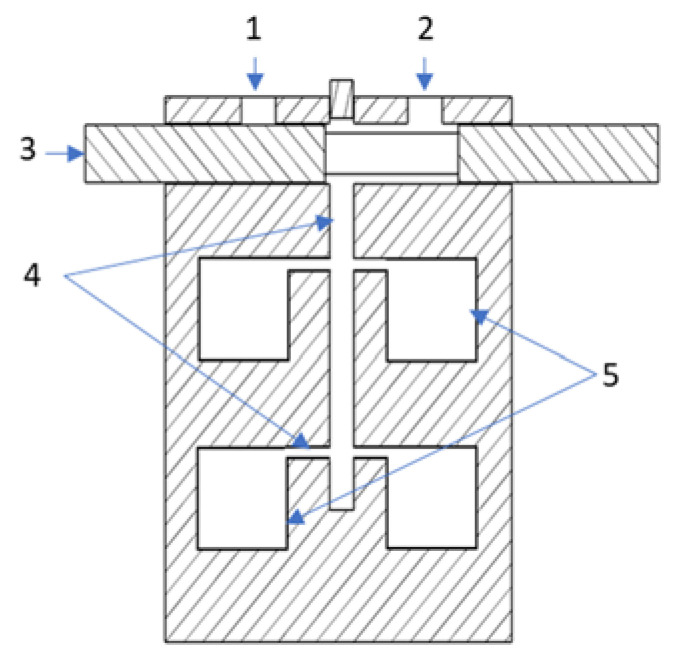
Three-way valve. 1. The inlet of reference/cleaning gas, 2. The inlet of test gas, 3. Moving part of the three-way valve, 4. Gas channels, 5. Sensor cells.

To control the flow of gas through the measuring chamber, a three-way valve was used. It was fixed in the corps of the measurement module next to the sensors chamber, as shown in Figure 4. The moving element, with a diameter of 3.4 mm and an overall length of 54 mm, was made of hardened steel and moved in a bronze sleeve. The position of the valve was controlled by TowerPro’s SG90 micro servo, TowerPro, Ponte Vedra, FL, USA with a power of 1.8 kg*cm, speed, 0.1 s/60°, and a weight of 9 g. The servo power supply voltage was 5 V.

The fit design ensured smooth operation of the valve while maintaining full tightness of the system. By making special channels in the moving part of the valve, it was possible to control the flow of gas from two different inputs. This allowed the sensors to be exposed to the test gas or the cleaning/reference gas. This solution guaranteed the small size of the pneumatic system and low power consumption. A current of 100 mA was consumed only for a period of 100 ms when changing the valve position. After that, the control servo remained inert and the servo motor control system consumed only 10 mA of current.

#### 2.2.3. Set of Sensors and Measurement Chamber

**Figure 5:**
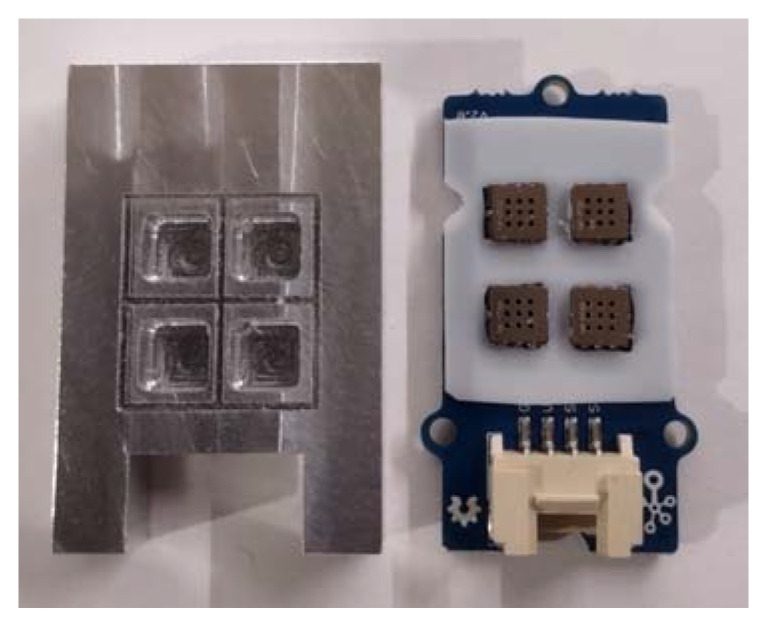
Measurement chamber (left) and Grove’s 42 × 24 mm four-channel gas sensor module (right).

Figure 3 shows the schema of the measurement platform. Its key component was Grove’s 42 × 24 mm four-channel gas sensor module, as shown in Figure 5 [18]. It is a compact device equipped with an independent microcontroller TM32F030, Seed Studio, Shenzhen, China which converts analog signals generated by semiconductor sensors into digital form. The sensor module communicated via an I2C interface supported by the microcontroller that controlled the sensor module. It was equipped with four independent semiconductor gas sensors GM-102B (NO2) [19], GM-302B (ethanol) [20], GM-502B (ethanol, formaldehyde, toluene, and other volatile organic compounds) [21], GM-702B (CO) [22] from Winsen Inc., Zhengzhou, China. They can detect various gases such as carbon monoxide, nitrogen dioxide, ethyl alcohol, volatile organic compounds, etc., but they featured cross-sensitivity. The sensors were manufactured using micro-electro-mechanical systems (MEMS) technology. They are characterized by long life, fast response time, small size, and high sensitivity 

The sensor module was equipped with a dedicated Teflon gasket, located between the printed circuit board (PCB) and the measurement chambers, to seal the pneumatic system.

**Figure 6:**
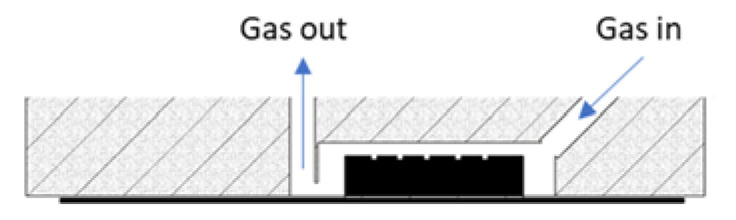
Gas flow through the sensor cell.

The chambers for the individual sensors were made of solid aluminum along with a built-in miniature three-way valve. It is a compact design in which gas flows through an internal 2 mm diameter channel system connecting the individual chambers to the three-way valve. The gas in the measuring chamber flowed from top to bottom, cutting across along the longest line, as shown in Figure 6. This organization of gas movement in the sensor cell and the small size of the cell allowed for a quick and complete exchange of the analyzed gas sample. Thanks to this, the device was characterized by a very short response time to changes in the composition of gas sampled by the measuring platform.

Outside the measurement system operating in the dynamic mode, the BME680 environmental sensor was located. The module is a combination of a total volatile organic compounds (TVOC) sensor, temperature sensor, humidity sensor, and barometer. Its primary task in our application was to measure atmospheric pressure for determining the current height of the measuring platform, relative to a reference point. The reference height can be set by the operator, controlling the operation of the measuring platform. During the tests, the height of the measurement platform was counted from the ground, at the drone’s launch site. 

The data sampling resolution of all sensors in the measurement system was set at 420 ms.

#### 2.2.4. An Air-Extracting Pump

The gas outlet from the measurement chamber was connected to a miniature air extraction pump, operated via the digital-analog converter (DAC) output of the microcontroller controlling the measurement platform. The pump used the model JSB1015091, weighing 8 g and measuring 8 × 10 × 15 mm equipped with an M20 motor with an operating voltage of 3V–5V DC. The input and output manifolds had an outer diameter of 3.2 mm with a flow diameter of 1 mm. The pump was made with diaphragm technology. It used the reciprocating motion of the diaphragm, generated by a DC motor to allow air to flow using appropriate check valves. A DC motor controller based on a BC337 transistor was used to enable smooth control of the air flowing through the measurement system via the DAC output of the microcontroller.

During testing, the gas flow through the gas sensing module was set to 11 l/h. The pump’s supply voltage was 3.3 V and it consumed 65 mA of current. The pump was attached by an aluminum adapter to a corps including a sensor chamber and a three-way valve.

#### 2.2.5. A Gas Transfer Line

The individual components of a sensing platform were connected by a gas transfer line prepared from tiny polytetrafluoroethylene (PTFE) tubes. The diagram of pneumatic connections is shown in Figure 3. A gas transfer line allowed the gas sensors to be exposed to the incoming air due to the operation of an air-extracting pump. The gas line was relatively short to avoid water condensation and gas adsorption. Elimination of surface chemistry phenomena during the presence of the gas in the inner tube was especially important in the case of gas sampling for VOCs measurement. For this reason, the system was cleaned with clean air after each measurement. The duration of an individual measurement was several minutes due to the drone flight time limit.

#### 2.2.6. Microcontroller

The gas-sensing platform was equipped with a small Seeeduino Xiao module with a 32-bit SAMD21G18 microcontroller based on the ARM Cortex-M0+ processor, which operates at up to 48 MHz. It features 256 kB of flash memory, 32 kB of static random-access memory (SRAM), and support for digital and analog interfaces. It is powered by 3.3 V and consumes a maximum of 20 mA of current. The module was used to control and manage the operation of the measurement platform, sensors, data acquisition, wireless transmission, and data processing, which were received via I2C buses using addresses and assigned to each device:

- SeeedStudio multi-channel gas sensor module (address 0 × 55);
- 4-in-1 BME680 multifunctional environmental sensor (address 0 × 76);
- DS1307 real-time clock (address 0 × 68).

**Figure 7:**
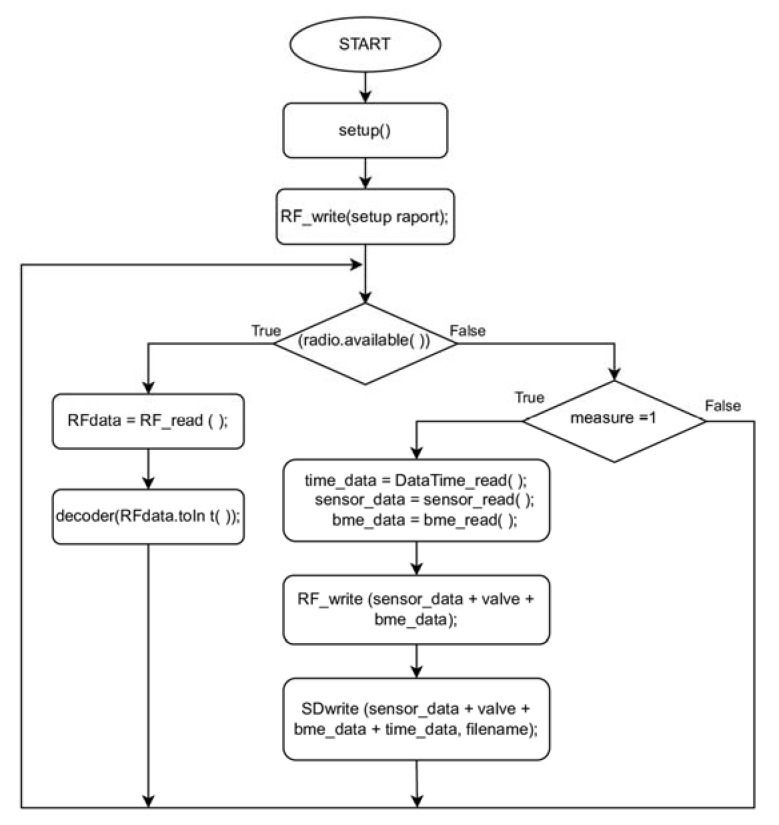
Algorithm of a program managing the operation of the measurement platform.

**Figure 8:**
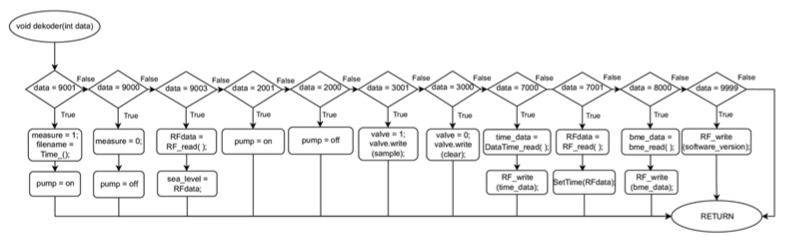
The process of interpreting the transmitted information and taking appropriate action.

Figure 7 shows the algorithm of the program that managed the operation of the measurement platform. After starting the device, the system configured the operating parameters of all modules on the measurement platform, informing the user via the wireless communication system of the device’s configuration status. The main loop of the algorithm started by checking the register of the RF receiver. When a command was received from the user, the system passed a value of integer type to the function decoder (RFdata.toIn t( )); this is shown in Figure 8 where the process of interpreting the transmitted information and taking appropriate action takes place:

- 9001–Change the parameter ‘measure’ (1- measure mode on and start the air pump, assign a value to the ’filename’ variable);
- 9000–Change the parameter ‘measure’ (0 - measure mode off and stop the air pump);
- 9003–Change the altitude above sea level, the command sets the altitude to zero at the current position;
- 2001–air pump on;
- 2000–air pump off;
- 3001–change the position of the three-way valve, the valve directs the air under test to the sensors chamber;
- 3000–changing the position of the three-way valve, the valve directs clean air from the activated carbon filter to the sensors chamber;
- 7000–sending the current date and time from the RTC module to the user;
- 7001–configuring the current date and time for the RTC module;
- 8000–sending the data from the BME680 sensor to the user;
- 9000–sending information about the current version of the measurement system to the user.

After receiving the 9001 command, the algorithm changed the value of the ‘measure’ register. This initiated the measurement procedure that collects information from the RTC, BME680, and multisensor module. After the data reading procedure was completed, the information from the sensors from the multisensor module, together with the value of the ‘valve’ register and the data from the BME sensor, was sent by radio, via the RF module, to the user. The next step was to save the sent information on the micro-SD card, adding at the end the ‘time–data’ variable specifying the current time and date. The data from each measurement process started and completed were saved in a separate file, the name of which specified the day and time the measurement started. The file name was specified by the ’filename’ variable, which was assigned the value of the current date each time a measurement process was started with the 9001 command.

### 2.3. Equipment for Lowering and Lifting the Measurement Module

The equipment for lowering and lifting the measurement platform played a critical role in the developed gas-sensing drone. This winch was designed as a compact and lightweight module. Its weight was 185 g. The module included a mechanism, which allowed it to quickly and easily lift the sensing platform, lower it, and keep it in a stable position. 

**Figure 9:**
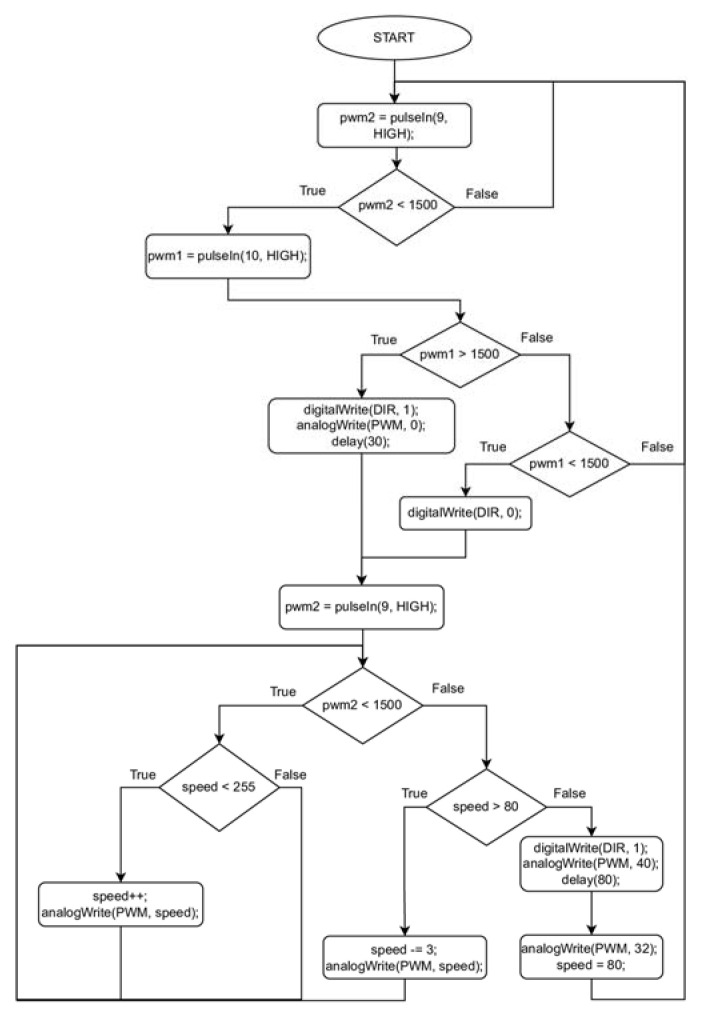
The algorithm of operation of the equipment for lowering and lifting the measurement module.

The equipment for lifting and lowering was equipped with a drum, which had an inner diameter of 30 mm, an external diameter of 40 mm, and a 12 mm working width. The drum was driven by a miniature electric motor that ran on DC electric power 6 V supplied by a battery. The motor was controlled by a microcontroller using a PWM signal. The motor converted the electrical energy into mechanical torque of the drum, consuming a maximum of 1 A of current at a maximum holding line winding speed of 580 rpm (which was 52 m/min at maximum speed). The engine was equipped with a 9.68:1 transmission. To stop the drum during sensing platform lowering, first, a current was applied to the DC motor, opposite to the lowering direction. After the drum was stopped, a blocking current of 280 mA was applied to the motor. A holding line was wound onto the drum. The line had a total length of about 25 m and a diameter of 0.3 mm. The length of the line allowed us to perform measurements, keeping a sufficient distance between the sensing platform and the drone. A small circuit board Cytron MD10C was used for operating the motor. The very precise speed control of the line motion was provided. The algorithm of operation of the equipment for lowering and lifting the measurement module is shown in Figure 9.

The apparatus for lowering and lifting the sensor platform was mounted on board the drone. The total weight of this module was 180 g. Its operation was controlled remotely through the drone controller.

### 2.4. Docking System

**Figure 10:**
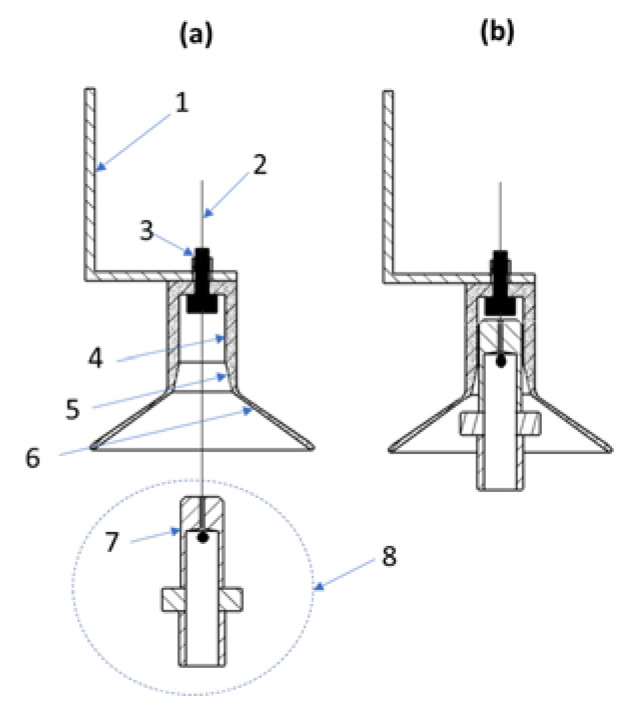
Docking system when (**a**) the measurement platform is undocked, and (**b**) the measurement platform is docked. (1) Fixing element; (2) Holding line 0.3 mm; (3) Hardened M3 bolt with 0.8 mm hole and nut; (4) cylindrical stabilizing bore (diameter 10.2 mm); (5) Guiding cup 30°; (6) Guiding cup 60°; (7) Cylindrical stabilizing bolt (diameter 10 mm); (8) Quick mounting system for the measurement platform.

To ensure optimal parameters and flight stability, a docking system was designed that connects the measurement platform with the drone and keeps it fixed during the flight, see Figure 1. The details of the mechanism are shown in Figure 10.

The docking system consisted of a corps and a detachable element. Both parts were made of polyoxymethylene (POM). This material ensured a high resistance and low weight of the system. The size of the docking system was 34 mm in length. It was attached to the drone (the body of the winch motor) with an aluminum element. To attach the docking system to this element a hardened screw was used, in which a hole was drilled. A line holding the measuring platform passed through the hole. Immobilization of the measuring platform during flight was achieved by embedding a cylindrical stabilizing bolt in the stabilizing bore. The docking system was equipped with a two-stage cup. It protected the holding line from damage when docking a swinging measurement platform. The cup consisted of two steps. The first step was made at an angle of 30°. It protected the holding line from damage when the measuring platform approached the docking system. The second step was made at an angle of 60°. The maximum diameter of the cup was 47 mm. The two-stage cup facilitated the embedding of the cylindrical stabilizing bolt in the stabilizing bore. The stabilizing bore was made to a length of 11 mm. On all edges of the docking system, radii were made and polished to minimize the likelihood of holding line damage when lowering or lifting the measuring platform. A clearance of 0.2 mm was left between the matching parts of the docking system. This allowed the measurement platform to be freely undocked from the drone by the force of gravity acting on the device being lowered. At the same time, they remained well-bonded and safe during transport. 

The docking system secured the drone’s flight by eliminating interference caused by swinging the platform on the holding line. It also enabled the emergency stabilization of the measurement platform in the event of excessive swinging during the measurement. The docking system was capable of immobilizing the platform under the drone during flight. Full protection of the holding line from abrasion or cutting was ensured by the special design of the docking system. 

### 2.5. Radio Communication

The measurement platform communicated with the user using a radio module equipped with an amplifier and antenna. The module’s design was based on the NRF24L01 chip, which has two-way communication on the 2.4 GHz band. It features a data transmission rate of up to 2 MB/s. Thanks to the module’s built-in amplifier, the chip had a power of 22 dBm, which allowed communication in open areas up to 1 km.

The user, communicating with the measurement platform, was equipped with a laptop with a dedicated GUI application for measurement process control.

To enable the computer to communicate with the measurement platform, it was necessary to connect, via USB interface, a dedicated transmitting and receiving device equipped with an antenna. The transmission device was based on the NRF24L01 chip with an amplifier, identical to in case of the measurement platform. It was operated by the Atmega328P microcontroller which communicated with the computer via a universal serial bus—universal asynchronous receiver and transmitter (USB-UART) converter.

## 3. Operation of the Gas-Sensing Drone

The operation of gas sensing drone included the following steps: (1) reference measurement; (2) start and flight to the target area; (3) achieving the location over the measurement point and hovering above it; (4) lowering the gas sensing platform; (5) gas sampling and the measurement of sensor responses to test gas (detection of air pollution); (6) lifting the gas sensing platform; (7) return flight to the dock location, and (8) sensor regeneration.

## 4. Test Measurements

### 4.1. Air Pollution Source

The developed gas-sensing drone was tested in field conditions. Open biomass burning was the kind of air pollution source chosen for test measurements. The open burning of tree branches, leaves, and the grass is commonly encountered in the world [23,24]. The associated smoke and emission of gaseous air pollutants cause irritation and numerous public health problems [25]. Hence, their detection is an important issue. In general, the open burning of unwanted materials is a serious risk to the environment and safety. The emissions from these sources are released directly into the air, at a low height. The dispersion of the released compounds is strongly affected by the local meteorological conditions. Therefore, stationary monitoring performed in fixed places is not capable of providing relevant information. The measurement instruments based on the UAV seem to be more adequate in such circumstances.

### 4.2. Experiments

The comparative experiment consisted of two measurement experiments, performed one after another. The measurement experiment focused on recording the response of the sensing drone to the air pollution released by the emission source when using (1) the sensing platform lowered from the drone, and (2) the sensing platform docked under the drone. In course of the entire measurement experiment, there were recorded signals of gas sensors, as well as results of measurements of air temperature, relative humidity, and sensing platform height above the ground.

(1) Measurement using a sensing platform lowered from the drone.

In this experiment, the relative position of the drone and gas sensing platform was not fixed, but it was purposefully changed. The experiment had the following stages: 1. sensors signal reference measurement—drone remained still at the starting location, on the ground. The sensing platform was set to realize the reference measurement mode. Stage duration was around 1 min. 2. Measurement of sensor signals to air pollution—the sensing platform was set to realize the measurement mode. Stage duration was about 1 min. 3. Take off and fly to the target area—drone took off and flew towards the target location, above the emission source. Stage duration was about 1 min. 4. Achieving the position over the measurement point and hovering above it—drone reached the position over the emission source and hovered above it for several seconds. The hovering height was several meters, depending on the experiment. The measurement mode remained on. 5. Lowering the gas sensing platform to the measurement point—the drone remained hovering at the height of several meters above the emission source. The sensing platform was undocked and lowered down to the measurement point, above the emission source at a vertical distance of approximately 20–70 cm from it. The measurement mode remained on. Stage duration was several seconds. 6. Detection of air pollution at the measurement point—drone remained hovering at the height of several meters above the emission source. The measurement platform hovered directly above the emission source, at a height of 20–70 cm above it. Air pollution measurement performed. Stage duration was several minutes. 7. Lifting the gas-sensing platform—drone remained hovering at the height of several meters above the emission source. The sensing platform was lifted vertically and docked to the drone. The measurement mode remained on. Stage duration was several seconds. 8. Return flight and landing at the starting location—drone flew towards its starting location and landed. The measurement mode remained on. Stage duration was about 1 min. 9. Sensors regeneration—after return, drone remained still at the starting position on the ground. Sensor cleaning was initiated.

(2) Measurement using a sensing platform docked under the drone

In this experiment, the position of the gas sensing platform under the drone was fixed and it was not changed. The experiment had the following stages: 1. to 4.—the same as in measurement experiment 1. 5. Descent to the measurement point—the drone descended vertically towards the emission source approaching it at the vertical distance of about 20–70 cm. The measurement mode remained on. Stage duration was several seconds. 6. Detection of air pollution at the measurement point—drone hovered at the attained vertical distance from the emission source. Air pollution measurement was performed. Stage duration was several minutes. 7. Ascent—drone flew upwards from the emission source and hovered at the height of several meters for a moment. The measurement mode remained on. Stage duration was several seconds. 8 and 9.—the same as in experiment 1. 

Two comparative experiments arranged in the field conditions were chosen for presentation in this work: Comparative Experiment 1 and Comparative Experiment 2.

(1) Comparative Experiment 1 (CE 1)

The source of air pollution was open wood burning. The burning was arranged in a garden grilling utensil. We used a small structure made of concrete, fitted with an open fireplace on the top. The fireplace was elevated about 1 m above the ground. Cut tree branches were used as fuel. A portion of the branches was burned during each measurement experiment.

**Figure 11:**
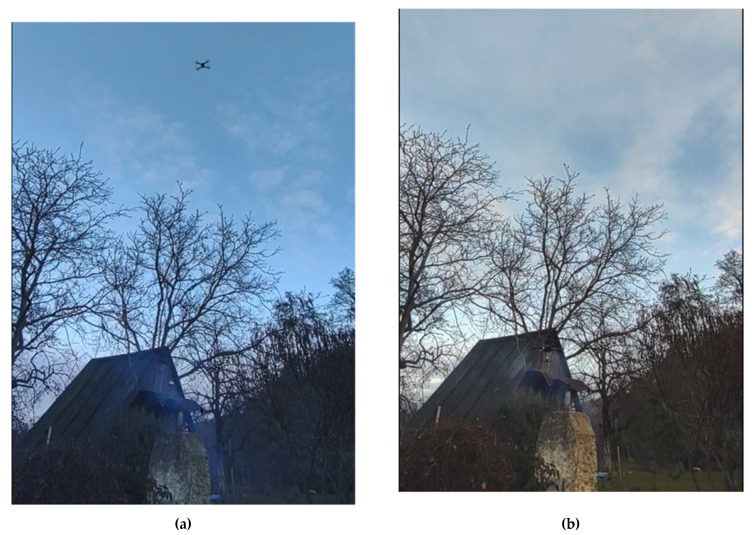
Comparative experiment CE1 (branches burning). The sensing platform was: (**a**) lowered from the drone, (**b**) docked under the drone.

The experiment was conducted in the hilly terrain, as shown in Figure 11, and in Video S1, see Supplementary Material. Nearby the emission source there was a hilltop, and in its immediate vicinity, there was a shelter and some trees. This kind of surrounding increased the turbulence of the natural airflow around the emission source. On the other hand, it provided shelter from the strong wind.

Measurements were performed on a bright autumn day. During the experiment, the air temperature was t = 7.8 ± 0.5 °C and the relative humidity of air was RH = 73 ± 2%. Hence, the meteorological conditions were very stable.

(2) Comparative Experiment 2 (CE 2)

The source of air pollution was touchwood burning. The burning was arranged in a smoker used by beekeepers. During experiments, the smoker was placed on the ground. A similar portion of touchwood was burned during each measurement experiment.

**Figure 12:**
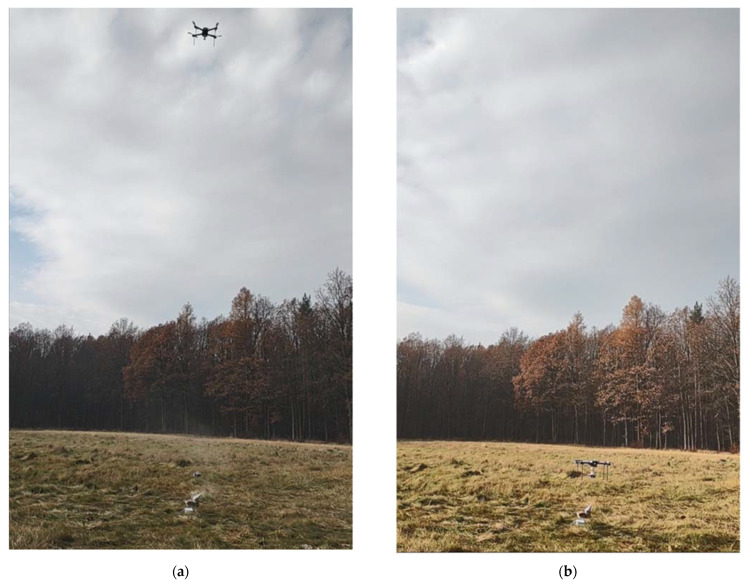
Comparative experiment CE2 (touchwood burning). The sensing platform was: (**a**) lowered from the drone, (**b**) docked under the drone.

The experiment was conducted on flat terrain, as shown in Figure 12, and in Video S2, see Supplementary material. Around the emission source, there was a large grassland. The forest started about 100 m away. This kind of surrounding resulted in considerable exposure to the wind.

Measurements were done on a bright autumn day. During the experiment, the air temperature was t = 12 ± 3.4 °C and the relative humidity of air was RH = 62 ± 9%. The meteorological conditions were unstable.

## 5. Results

**Figure 13:**
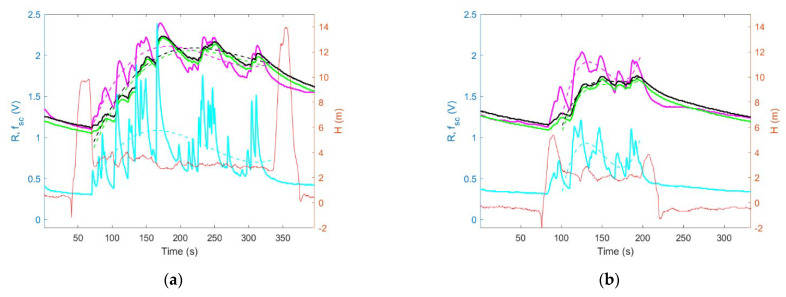
Results of measurements performed by the sensing drone during experiment CE1, when the sensing platform was: (**a**) lowered from the drone, (**b**) docked under the drone. Gas sensor signals are shown using solid lines: GM102B (magenta), GM302B (black), GM502B (green), and GM702B (cyan). The systematic components of sensor signals are shown using dashed lines, respectively. A solid red line indicates the sensing platform height above the ground.

**Figure 14:**
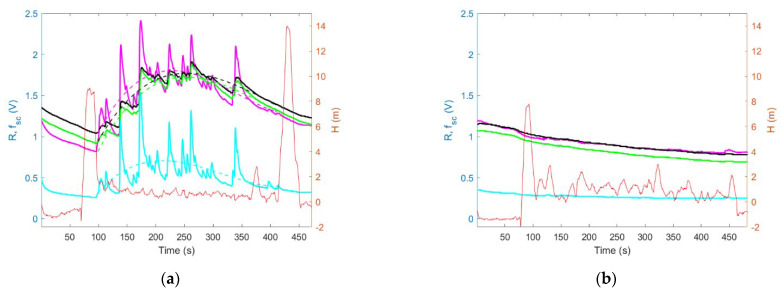
Results of measurements performed by the sensing drone during experiment CE2, when the sensing platform was: (**a**) lowered from the drone, (**b**) docked under the drone. Gas sensor signals are shown using solid lines: GM102B (magenta), GM302B (black), GM502B (green), and GM702B (cyan). The systematic components of sensor signals are shown using dashed lines, respectively. A solid red line indicates the sensing platform height above the ground.

The sensing drone was tested in field conditions. Figure 13 and Figure 14 present the results of preliminary comparative experiments CE1 and CE2, respectively. Left panels in Figure 13 and Figure 14 display sensor signals recorded by the sensing platform lowered down to the measurement point, while the drone itself hovered several meters above it. Right panels in Figure 13 and Figure 14 display sensor signals recorded by the sensing platform, which was docked under the drone while operating at the measurement point.

Sensor signal represents a series of sensor responses recorded at the subsequent time points. Therefore, it may be viewed as a time series. Any time series may be understood as the aggregate of systematic and non-systematic components. The systematic components of the time series have consistency, and they can be described and modeled, while non-systematic components are represented by the random variation in the time series. We assumed that the sensor signal is the additive combination of the two. It was proposed to model the systematic components using the third-order polynomial. The non-systematic components of the sensor signal were determined as the difference between the sensor signal and the model of the systematic components.

In Figure 12 and Figure 13, the solid lines, except for the red one, represent the signals (R) of gas sensors GM102B, GM302B, GM502B, and GM702B. Dashed lines show the systematic components (f_sc_) of the gas sensor signals which were recorded while the sensing device operated at the measurement point. The red line in Figure 12 and Figure 13 shows the height of the sensing platform above the ground in course of experiments.

**Figure 15:**
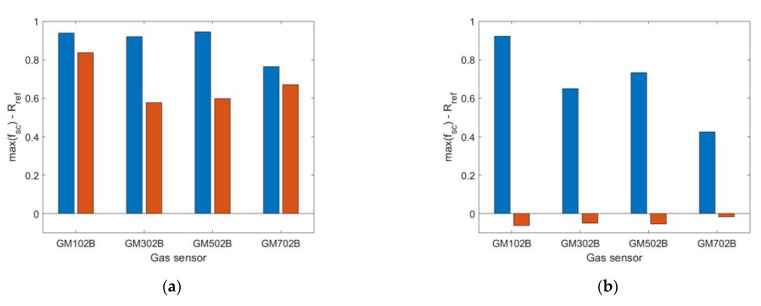
The magnitude of sensing drone response to air pollution when the sensing platform was lowered to the measurement point (blue bar) and when it was docked under the drone (red bar) during measurement, based on experiments: (**a**) CE1 (branches burning), and (**b**) CE2 (touchwood burning).

Figure 15 shows the magnitude of sensing drone response to air pollution at the measurement point. It was defined as the difference between the maximum value of the systematic component of the sensor signal recorded during exposure at the measurement point and the sensor signal reference value (R_ref_). The sensor signal reference value was the average of the sensor’s responses recorded during the last five seconds of the reference measurement. This period was chosen arbitrarily. Blue bars in Figure 15 represent the magnitude of the sensing drone’s response to air pollution when the sensing platform was lowered down to the measurement point, while the drone hovered several meters above it. Orange bars in Figure 15 represent the magnitude of the sensing drone’s response to air pollution when the sensing platform was docked under the drone while operating at the measurement point.

**Figure 16:**
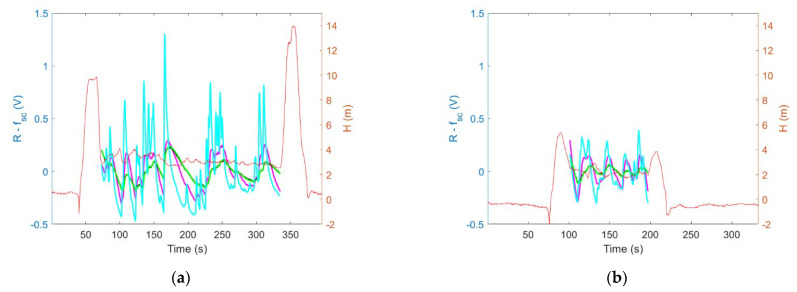
The variation of gas sensor signals recorded during experiment CE1 when the sensing platform was: (**a**) lowered from the drone, (**b**) docked under the drone. Gas sensor signals without systematic components are shown using the following colors: GM102B (magenta), GM302B (black), GM502B (green), and GM702B (cyan). The red line indicates the sensing platform height above the ground.

**Figure 17:**
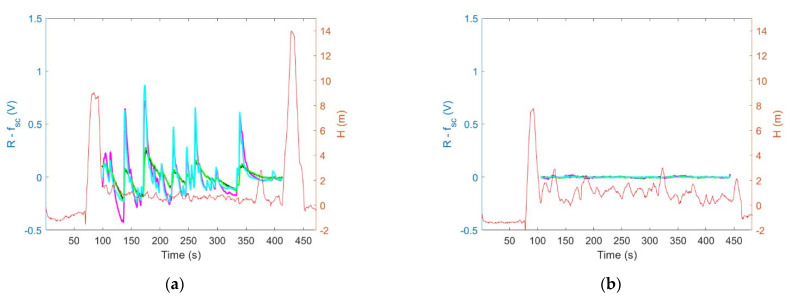
The variation of gas sensor signals recorded during experiment CE2 when the sensing platform was: (**a**) lowered from the drone, (**b**) docked under the drone. Gas sensor signals without systematic components are shown using the following colors: GM102B (magenta), GM302B (black), GM502B (green), and GM702B (cyan). The red line indicates the sensing platform height above the ground.

Figure 16 and Figure 17 show the gas sensor signals variation during the field experiments CE1 and CE2, respectively. The sensor signal change was obtained by subtracting the systematic component from the sensor signal (R-f_sc_). The left panels in Figure 16 and Figure 17 display the variation of sensor signals recorded by the sensing platform lowered down to the measurement point while the drone itself hovered several meters above it. The right panels in Figure 16 and Figure 17 display the variation of sensor signals recorded by the sensing platform, which was docked under the drone while operating at the measurement point.

**Figure 18:**
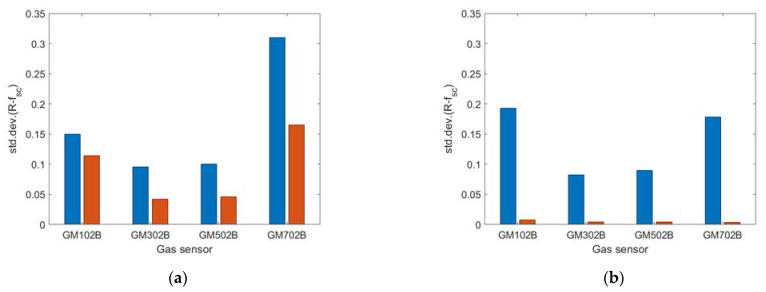
The magnitude of sensor signals variation due to exposure to air pollution when the sensing platform was lowered to the measurement point (blue bar) and when it was docked under the drone (red bar) during measurement, based on experiments: (**a**) CE1 (branches burning), and (**b**) CE2 (touchwood burning).

Figure 18 shows the magnitude of sensing drone response variation caused by exposure to air pollution at the measurement point. It was defined as the standard deviation of the differences between the sensor signal and its systematic component in the period of exposure to air pollution at the measurement point. The blue bars in Figure 18 represent the magnitude of sensing drone response variation when the sensing platform was lowered down to the measurement point, while the drone hovered several meters above it. Orange bars in Figure 18 represent the magnitude of sensing drone response variation when the sensing platform was docked under the drone while operating at the measurement point.

Our preliminary field experiments showed that the sensitivity of the sensing drone was impaired when air pollution was measured using a sensing platform docked under the drone. Greater sensitivity of measurement was attained when the sensing platform was lowered down to the measurement point while UAV remained to hover above it, at some distance.

Based on the comparison of Figure 13a with Figure 13b, and as shown in Figure 14a, the magnitude of sensing drone response to air pollution was greater when the sensing platform was lowered down to the measurement point (Figure 13a and Figure 15a (blue bars)) as compared with the case when the sensing platform was docked under the drone while performing air pollution measurements (Figure 13b and Figure 15a (orange bars)).

Moreover, based on the comparison of Figure 14a with Figure 14b, and as shown in Figure 15b, the measurement with the sensing platform docked under the drone resulted in not detecting any air pollution (Figure 14b and Figure 15b (orange bars)), while the measurement performed using sensing platform lowered down to the measurement point (Figure 14a and Figure 15a (blue bars)) revealed substantial contamination.

The performed experiments showed that the resolution of the sensing drone was impaired when the sensing platform was docked under the drone while measuring air pollution. Greater measurement resolution was attained when the sensing platform was lowered down to the measurement point, while UAV remained hovering above it, at some distance.

Based on the comparison of Figure 16a with Figure 16b, and as shown in Figure 18a, the magnitude of sensing drone response variation due to exposure to air pollution was greater when the sensing platform was lowered down to the measurement point (Figure 16a and Figure 18a (blue bars)) as compared with the case when the sensing platform was docked under the drone while performing air pollution measurements (Figure 16b and Figure 18a (orange bars)).

Moreover, based on the comparison of Figure 17a with Figure 17b, and as shown in Figure 18b, the air pollution measurement performed using the sensing platform docked under the drone resulted in not detecting the variation of air pollution (Figure 17b and Figure 18b (orange bars)), while the measurement performed using sensing platform lowered down to the measurement point (Figure 17a and Figure 18a (blue bars)) revealed its substantial variation.

## 6. Discussion

Currently, the UAV seem to be the most convenient means of transportation for measurement systems, which makes them mobile and capable of performing measurements in the air, in various locations, and at different distances from the ground. However, the propulsion system of the drone induces airflow and air turbulence in drone proximity. Due to this, the conditions of gas dispersion are changed, and the initial concentration field is disturbed in the zone where the drone appears. Therefore, if the measurement is performed in the zone of the drone’s impact, the obtained result does not reflect the actual pollution level at the measurement point. To assure reliable measurement, it is necessary to maximize the detection performance against rotor flow effects at the measurement point. In practice, this means measuring in the zone where the air turbulence caused by the drone is at its lowest. This involves placing a sensor, a measuring device, or just an air intake, to take a gas sample in this zone. At the same time, it is necessary to ensure the stability of the drone both during flight and hovering. For these reasons, the most convenient location would be on board a drone. A considerable research effort focused on identifying the zones of minimum rotor flow effects close to the drone body. As the potential places for sensor/sensor device/air intake, the bottom of the drone corps [3,4,10], its top [10,12], and its sides, mostly at the front [2,10], were considered. However, locations at some distance from the body of the drone, such as on the rotor arm(s) [9,10] or even farther away, which involves the use of a special boom [15], are also taken into consideration.

The presented sensing drone with the lowered and lifted multi-sensor measurement platform was designed to overcome the problem of disturbance of the pollution concentration field caused by the drone at the measurement point and assure a reliable measurement. For construction reasons, in particular the ability to ensure drone stabilization, it seems most convenient to place the measuring device directly under the body of the drone or on top of it. However, the literature reports indicate that both of these positions are unfavorable. Regarding top location, it was shown in [10] that sensors mounted on the top of the drone showed minimum sensitivity, compared with frontal, bottom, and rotor locations. The limited sensitivity in the top location was linked to the absence of upward airflow associated with rotor movement, and the associated limited pollution transport from the space around the drone to this particular point of sensor exposure. The zone above the drone corps was not significantly affected by the rotor flow effect. This fact causes the top location of the sensor to be used in applications aimed at preparing vertical profiles of various air parameters in the atmosphere. However, to assure reliability, it is requested that the measurement is performed on a vertical flight path, during ascent. Based on [12] measurements conducted in a vertical flight path, using the measurement system mounted on the top of the drone shows promise compared with ground truth references data regarding PM2.5, temperature, and humidity. In environmental applications aimed at the determination of pollution in various locations and circumstances, the request for vertical ascending flight is a serious limitation.

Regarding the bottom location of the measurement device, it was shown in [15] that the detection of VOCs failed when the air was sampled on the bottom of the drone. The unsuccessful extraction of VOCs was attributed to the downward airflow drawn from rotating propellers. When applying a telescoping shaft to extend the sampling range beyond the strong downward stream that flows across the propellers, considerable concentrations of toluene, benzene, and p-xylene were detected. Concentration decrease under the drone was also reported in [9]—based on measurements and in [10]—based on simulations. In [6,8] the downwash effect was studied in detail, using simulation. It was shown that, regardless of the hovering height, the airflow entered the region of rotors from above and exited downward. Additionally, it the so-called ground effect was identified. Namely, the downflow was reflected from the ground creating a turbulence zone. It was shown that, by increasing the hovering height, the proportion of space occupied by the turbulence decreased. When the hovering height was boundless, the entire flow field moved downward without turbulence and the flow velocity decreased to zero. The operational hovering height appropriate for the drone used in [8] was identified as 3 m. 

The above problems were experienced by the constructions based on the assumption that the measuring device was permanently attached to the drone, in its vicinity. Our concept was based on the idea of periodically changing their positions relative to each other. In particular, we proposed that the device should remain docked to the drone for the time of transport, and the distance between them should be as large as possible for the time of measurement. The design of our sensing drone with the lowered and lifted measurement platform is strongly supported by the results presented in [6,8,9,10,15]. The developed solution made it possible to carry out measurements at a distance of several meters from the drone, which hovered high above the measurement point. Thanks to this, the impact of downward flow on the measurement result was minimized. In addition, it was possible to measure close to the ground surface while avoiding the ground effect. The small size and low weight of the sensing device made the turbulence caused by its lowering, lifting, and potentially swing and rotation small.

These advantages of our sensing drone with the lowered and lifted measurement platform were confirmed in field experiments, which focused on measurements of air pollution caused by open burning. During field tests, we observed that the proposed sensing drone minimized the effect of the pollution concertation decreased at the measurement point, as a result of the downward airflow induced by drone rotors. Such an effect was experienced when the measurement platform was docked directly under the drone (Figure 11b). In this case, the magnitude of sensor responses, as shown in Figure 13b and Figure 15a (orange bars), was smaller than in Figure 13a and Figure 15a (blue bars), which display the measurements results of the sensing platform lowered from the drone (Figure 11a), and operating at the measurement point, at the distance of more than several meters from the UAV.

In the course of the field experiments, we also observed that the developed sensing drone reduced pollution concentration decrease caused by downwash and enhanced by the ground effect. Such an effect was observed when the measurement was performed very close to the ground by the measurement platform docked directly under the drone (Figure 12b). In this case, no pollution was detected, as shown by the magnitude of sensor responses in Figure 14b and Figure 15b (orange bars) compared with Figure 14a and Figure 15b (blue bars), which present the results of measurements when the sensing platform was lowered from the drone (Figure 12a).

Based on tests performed in real conditions, we additionally observed that the presented sensing solution limited pollution concertation field flattening below the drone, caused by the rotor-induced turbulence. Such an effect was observed when the measurement platform was docked directly under the drone (Figure 11b). In this case, the magnitude of sensor responses variation, as shown in Figure 16b and Figure 18a (orange bars), was smaller than in Figure 16a and Figure 18a (blue bars) which refer to the measurements performed by the sensing platform lowered from the drone to the measurement point (Figure 11a). In the case of very high air turbulence associated with ground effect, the measurement platform docked under the drone (Figure 12b) could present the concertation field as uniform, as displayed in Figure 17b and Figure 18b (orange bars), while in reality it was not uniform, as shown by the nonzero magnitude of sensor responses variation in Figure 17a and Figure 18b (blue bars).

The proposed solution of the sensing drone could raise concerns regarding drone stability, in particular during measurement platform lowering, lifting, and at the time of drone hovering while the sensing module remains lowered, several meters below the drone. During field tests, even in adverse weather conditions as well as in case of lack of accurate drone positioning due to a weak GPS signal, there was little swinging or rotational movement of the measurement platform. The maximum diameter of the circle, in which the measurement platform made a swinging motion, was about 4.5 cm. Despite such a sway, the pilot had full control over the drone, which did not lose its stability. The low weight of the measurement platform did not significantly affect the drone’s operation and the Pixhawk system’s gyroscope stabilized the flight without problems. During all tests of the measurement system and its earlier prototypes, the drone’s stability was not affected and there was no dangerous situation leading to loss of control of the drone. However, if there appears a considerable rotational or swinging movement of the platform, the system allows for its stabilization. This may be achieved within a maximum of 2 min. The respective action involves lifting the measurement platform, stabilizing it through the docking system, and then lowering it back to the measurement location.

## 7. Conclusions

The authors introduced a novel sensing drone for in-site measurements of air quality parameters, in field conditions. The preliminary tests demonstrated the proper operation of the developed system. Although more tests are needed, the obtained results have already shown that, with this low-cost construction, the interference with the measurement from the air turbulence generated by drone propellers is minimized. The test results confirm that the developed gas-sensing drone offers rapid detection of air pollutants with high measurement sensitivity and resolution in specific places. It is also possible to perform profiling of air pollution, e.g., the vertical distribution of the air pollutants concentration in the atmosphere using this measurement solution.

The presented technology may be an important step in developing effective mobile unmanned aerial tools that allow reaching dangerous places with poor accessibility and performing measurements there in an economical way, while keeping the operator at a safe distance. Various measurement scenarios are possible, e.g., detection of air pollution during chemical release events, post-disaster operations, and periodic monitoring of “hot zones”.

## 8. Patents

Szczurek Andrzej; Gonstał Dawid and Maciejewska Monika. Unmanned mobile measurement system for chemical measurements with high spatial resolution in air. Patent application no. P 439593, from 22 November 2021.

## Data Availability

Measurement data access restrictions apply.

## Supplementary Materials

The following supporting information can be downloaded at: https://youtu.be/4hJjpy1R3BY, Video S1: Comparative experiment CE1; https://youtu.be/WBgt0RwQYQI, Video S2: Comparative experiment CE2.
